# A hospital based surveillance of metallo-beta-lactamase producing gram negative bacteria in Nepal by imipenem-EDTA disk method

**DOI:** 10.1186/s13104-017-2640-7

**Published:** 2017-07-25

**Authors:** Pratigya Thapa, Dinesh Bhandari, Dhiraj Shrestha, Hiramani Parajuli, Prakash Chaudhary, Jyoti Amatya, Ritu Amatya

**Affiliations:** 1 Department of Microbiology, Trichandra Multiple College, Ghantaghar, Kathmandu, Nepal; 2 Public Health Research Laboratory, Institute of Medicine, Tribhuvan University Teaching Hospital, Maharajgunj, Kathmandu, Nepal; 3 Department of Microbiology, Nepal Medical College and Teaching Hospital, Kathmandu University, Dhulikhel, Nepal

**Keywords:** Metallo-beta-lactamase, Surveillance, Ceftazidime-resistant Gram-negative rods, Antimicrobial resistance

## Abstract

**Background:**

A rising threat of the rapid spread of acquired metallo-beta-lactamases (MBLs) among major Gram-negative pathogens is a matter of public health concern worldwide. Hence, for a low income nation like Nepal, surveillance data on MBL producing clinical isolates via a cost effective technique is necessary to prevent their dissemination as well as formulation and regulation of antimicrobial stewardship policy.

**Methods:**

The prospective study was conducted at Nepal Medical College, Kathmandu from May to October, 2014 to assess the prevalence of MBL production among ceftazidime-resistant Gram-negative rods (GNRs) isolates. The samples were processed according to standard microbiological procedure following the Manual of clinical Microbiology. Isolated GNRs were subjected to susceptibility testing against the selected panel of antibiotics by Kirby- Bauer disc diffusion method and interpretation made in conformity with the Clinical and Laboratory Standards Institute (CLSI) guidelines. Ceftazidime-resistant isolates were subjected to the detection of MBL production by imipenem—EDTA combined disc (CD) method.

**Results:**

Among the Gram-negative isolates, 5.80% (21/362) were found to be MBL positive with *Acinetobacter* spp. showing the highest prevalence i.e. 85.71% (18/21), followed by *P. aeruginosa* i.e. 14.29% (3/21). None of the other cefazidime resistant gram negative bacteria tested were found to be positive for MBL production with all the positive isolates determined to be Multidrug resistant (MDR) strains.

**Conclusion:**

This study demonstrated a higher rate of resistance among *P. aeruginosa* and *Acinetobacter* spp. to a wide variety of antibiotic categories with an additional burden of MBL production within them, warranting a need for strict surveillance and rapid detection of MBL production among the GNRs.

## Background

Metallo-beta-lactamase (MBL) activity has emerged as one of the most feared resistance mechanisms because of its ability to hydrolyze virtually all beta-lactams, including carbapenems. However, MBLs are unable to hydrolyze monobactams. Based on the molecular studies, carbapenemases i.e., enzymes hydrolyzing carbapenems are classified into four groups: A, B, C and D. Metallo-beta-lactamases belong to Amber class B type of beta-lactamase and act on a broad spectrum of substrates including penicillins, cephalosporins, and carbapenems [1]. MBL producing *Pseudomonas aeruginosa* was first reported in Japan in 1991, since then it has been reported in various parts of the world including Asia, Europe, Australia, South America, and North America [2–9]. The detection of MBL-producing Gram-negative bacilli is crucial to control the spread of resistance and for the optimal treatment of patients, particularly the critically ill and hospitalized patients [10].

In context of Nepal data on the burden of MBL producing Gram negative bacteria is limited [15]. Since, the knowledge about the types of enzymes that may be present can serve to guide the infectious disease physician toward choosing appropriate therapy without the need for extensive secondary testing, detection of MBL producing isolates is of paramount importance in clinical setting. Given the background, this epidemiological study was designed to generate updated information on the burden of metallo-beta-lactamase producing Gram negative bacteria from a tertiary care hospital in Nepal so that an effective antimicrobial stewardship policy can be formulated and implemented to circumvent the rising threat of antimicrobial resistance.

## Methods

### Study setting, design and study population

The prospective study was conducted at Nepal Medical College, Kathmandu from May to October 2014 to assess the prevalence of metallo-beta-lactamase production among the ceftazidime-resistant Gram-negative rods (GNRs) isolated from different clinical samples. A total of 4765 different clinical samples (sputum, pus, tracheal secretion, bronchial secretion, urine, and body fluids like CSF and peritoneal fluid) from patients of all age groups received in the microbiology laboratory for routine examination and culture, during the study period of six months were included in the study.

### Laboratory processing of the samples

All the sample specimens were processed by standard microbiological operating procedure for isolation and identification of microorganisms following the Manual of clinical microbiology [11]. Briefly, the samples were inoculated in routine culture media (blood agar, MacConkey agar, chocolate agar) [Hi media Laboratory Limited, Mumbai, India, LOT 0000137031], subjected for microscopic examination as Gram’s stained preparation and inoculums from culture plates tested in biochemical media for identification of the bacteria based upon their morphology, cultural characteristics and biochemical properties, in compliance with Manual of Clinical Microbiology [11].

### Antimicrobial susceptibility testing

Antibiotic susceptibility test of all the clinical isolates to antibiotics from various categories (supplier: Hi media Laboratory Limited, Mumbai, India) was performed by Kirby-Bauer disk diffusion method and interpretation of the results was made in compliance with CLSI guidelines [12]. Control strains of *P. aeruginosa* ATCC 27853, and *E. coli* ATCC 25922 were used in parallel as a part of quality control as well as for validation of the test performed. We considered the isolates resistant to at least three classes of first-line antimicrobial agents as the MDR strains [13].

### Detection of metallo-beta-lactamase producing strain

In this study phenotypic detection method as described below was followed for the detection of MBL isolates.

### Screening test

The isolates were subjected for MBL detection when the zone of inhibition (ZOI) for ceftazidime (CAZ) (30 µg) was <18 mm. The sensitivity or resistance pattern to imipenem (IPM) (10 μg) and/or meropenem (MEM) (10 μg) were not considered for MBL detection as bacteria might harbor “hidden MBL”. Thus to ascertain not a single isolate carrying hidden MBL is missed, we used ceftazidime resistance as the screening tool. A suspension of bacteria equivalent to 1:10 dilution of 0.5 McFarland were used to prepare a lawn culture in Muller Hinton agar and subsequent application of the antibiotic discs was carried out [14, 15].

### Combined disc (CD) method

Two IPM disks (10 µg), one containing 10 µl of 0.5 M (750 µg) anhydrous ethylenediamine-tetraacetic acid (EDTA) and the other without EDTA were placed 25 mm apart (center to center). An increase in zone diameter of ≥7 mm around the IPM-EDTA disk compared to that of the IPM disk alone was considered positive for MBL [16].

## Results

A total of 4765 different clinical samples (urine, pus, body fluids and sputum) were included from patients admitted or attending outdoor patient (OPD) during the study period of 6 months from May to November 2014. Out of them 664 (13.9%) samples were growth positive, among which 362 (54.5%) were Gram-negative isolates with *E. coli* being the most prevalent i.e., 199 (54.9%). Out of the total 362 Gram negative isolates, 196 (54%) samples were from the outdoor patients (OPD) and the remaining 166 (46%) samples were from the indoor (hospital admitted) patients. MBL was detected in 21 (5.8%) of the culture positive Gram-negative bacteria (Table 3).

### Distribution of isolates in different clinical samples

*Escherichia coli* from urine was the most prevalent isolate i.e., 179 (68.1%) and *Proteus vulgaris, Providencia* spp. and *Serratia* spp. were the least prevalent isolates i.e., 1 (0.2%) (Table 1).

**Table 1:** Overall distribution of isolates in different clinical samples

Isolates	Samples				
	Urine	Pus	Sputum	Body fluid	Total
*E. coli*	179 (68.1)	10 (2.7)	4 (1.1)	6 (1.6)	199 (54.9)
*P. aeruginosa*	27 (10.3)	5 (1.3)	16 (4.4)	6 (1.6)	54 (14.9)
*A. calcoaceticus baumanii* Complex	21 (8)	21 (5.8)	10 (2.7)	6 (1.6)	58 (16)
*K. pneumonia*	25 (9.5)	1 (0.2)	4 (1.1)	1 (0.2)	31 (8.5)
*Enterobacter* spp.	1 (0.4)	1 (0.2)	1 (0.2)	0 (0)	3 (0.8)
*P. mirabilis*	4 (1.5)	1 (0.2)	0 (0)	0 (0)	5 (1.3)
*P. vulgaris*	1 (0.4)	0 (0)	0 (0)	0 (0)	1 (0.2)
*Citrobacter* spp.	4 (1.5)	2 (0.5)	3 (0.8)	0 (0)	9 (2.4)
*Serratia* spp.	1 (0.4)	0 (0)	0 (0)	0 (0)	1 (0.2)
*Providencia* spp.	0 (0)	1 (0.2)	0 (0)	0 (0)	1 (0.2)
Total	263 (100)	42 (11.6)	38 (10.5)	19 (5.2)	362 (100)

### Antibiotic susceptibility pattern of the isolates

Out of the 362 Gram negative bacterial isolates tested, 159 (43.92%) *Enterobacteriaceae* members were found to be resistant to ceftazidime. Among the ceftazidime resistant *Enterobacteriaceae* members, the most effective antibiotics was nitrofurantoin i.e. 91 (91%) followed by amikacin i.e. 85 (85%). All the ceftazidime resistant *Enterobacteriaceae* members were also resistant to ampicillin (Table 2). Meanwhile, among the total *P. aeruginosa*, and *Acinetobacter calcoaceticus baumanii* complex isolates, 44.4 and 60.3%, respectively were found to be resistant against ceftazidime. Polymixin B and tigeycline were found to be the most effective drug among the cefatzidime resistance *P. aerugenosa* and *Acinetobacter* spp. respectively, as shown in Table 2.

**Table 2:** Antibiotic susceptibility pattern of the ceftazidime resistant Gram negative isolates (N = 159)

Antibiotics	Enterobacteriaceae family (N = 100)			*P. aeruginosa* (N = 24)			*Acinetobactercalcoac eticusbaumanii* complex (N = 35)		
	S (%)	I (%)	R (%)	S (%)	I (%)	R (%)	S (%)	I (%)	R (%)
Amikacin	85	0	15	75	0	25	31.4	2.8	65.7
Ampicillin	0	0	100	NT	NT	NT	NT	NT	NT
Ampicillin–sulbactam	NT	NT	NT	NT	NT	NT	31.4	2.8	65.7
Aztreonam	NT	NT	NT	62.5	4.1	33.3	NT	NT	NT
Cefepime	NT	NT	NT	NT	NT	NT	11.4	0	88.5
Cefotaxime	11	0	89	NT	NT	NT	11.4	0	88.5
Ciprofloxacin	21	3	76	45.8	0	54.1	14.2	2.8	82.8
Co-trimoxazole	28	0	72	NT	NT	NT	31.4	2.8	65.7
Doxycycline	NT	NT	NT	NT	NT	NT	34.2	2.8	62.8
Gentamicin	34	1	65	54.1	0	45.8	37.1	0	62.8
Imipenem	NT	NT	NT	91.6	0	8.3	68.5	5.7	25.7
Nitrofurantoin	91	0	9	NT	NT	NT	NT	NT	NT
Ofloxacin	22	0	78	48	0	54.1	NT	NT	NT
Piperacillin	NT	NT	NT	41.6	0	58.3	NT	NT	NT
Piperacillin–tazobactam	NT	NT	NT	79.1	0	20.8	37.1	0	62.8
Polymixin B	NT	NT	NT	100	0	0	NT	NT	NT
Tigecycline	NT	NT	NT	NT	NT	NT	85.7	0	14.2

*S* sensitive, *I* intermediate, *R* resistant, *NT* not tested

A total of 208 (57.5%) isolates were found to be MDR with most of them being *E. coli* i.e., 113 (56.8%) followed by *Acinetobacter* spp. i.e., 82.8% and *P. aerugenosa* i.e., 44.4% (Table 3).

**Table 3:** Multi drug resistance patterns and metallo-beta-lactamase production in the isolates

Isolates	MDR positive	MBL positive	Total
*E. coli*	113 (56.8)	0 (0)	199
*P. aeruginosa*	24 (44.4)	3 (5.6)	54
*A. calcoaceticus baumanii* complex	48 (82.8)	18 (31)	58
*K. pneumonia*	12 (38.7)	0 (0)	31
*Enterobacter* spp.	2 (66.7)	0 (0)	3
*P. mirabilis*	2 (40)	0 (0)	5
*P. vulgaris*	1 (100)	0 (0)	1
*Citrobacter* spp.	6 (66.7)	0 (0)	9
*Serratia* spp.	0 (0)	0 (0)	1
*Providencia* spp.	0 (0)	0 (0)	1
Total	208 (57.5)	21 (5.8)	362

### Distribution of the metallo-beta-lactamase producing organisms

Among the 362 Gram negative bacteria isolated, 21/362 (5.8%) were metallo-beta-lactamase producers. Among the MBL positive bacteria, the prevalence of *A. calcoaceticus baumanii* complex was the highest i.e., 18 (85.7%), followed by *P. aeruginosa* i.e., 3 (14.2%). None of the other Gram negative isolates were found to be metallo-beta-lactamase producers during the study (Table 3). Most of the MBL positive isolates were recovered from urine and pus i.e. 7 (33.3%), each and the least from body fluid i.e., 3 (14.2%) (Table 4).

**Table 4:** Distribution of metallo-beta-lactamase producers in different clinical samples

Samples	MBL positive				MBL negative			
	*A. calcoaceticus baumanii*	*P. aeruginosa*	Other Gram negative organisms	Total	*A. calcoaceticus baumanii*	*P. aeruginosa*	Other Gram negative organisms	Total
Urine	4	3	0	7	17	24	215	256
Pus	7	0	0	7	14	5	16	35
Sputum	4	0	0	4	6	16	12	34
Body fluids	3	0	0	3	3	6	7	16
Total	18	3	0	21	40	51	250	341

## Discussion

Metallo-beta-lactamases are a large and diverse group of beta-lactamases that are now disseminating on mobile genetic elements among clinically important Gram-negative pathogens, limiting treatment options for life-threatening infections [1, 17, 18]. Infection with the metallo-beta-lactamase (MBLs) producing organisms are associated with higher rates of mortality, morbidity, and health care costs [19]. In any nosocomial setting, carbapenems are used as the last resort for treatment of MDR Gram-negative bacterial infection. However, since last 15 years, acquired resistance to this life saving antimicrobial has been increasingly reported not only in *P. aeruginosa* and *Acinetobacter* spp. but also among members of *Enterobacteriaceae* which is mainly mediated by *Klebsiella pneumoniae* carbapenemases (KPC) [20].

In this study, among the 362 Gram-negative isolates recovered, 5.8% were found to be MBL producer which is higher compared to the prevalence rate of 1.3% reported from a similar study conducted in Nepal during 2012 that reported MBL producers from samples of lower respiratory tract (LRT) infection cases [15]. The findings of current study suggest that there is a continuous proliferation of MBL producers in Nepal. During the surveillance period of 6 months, only *P. aeruginosa* and *Acinetobacter* spp. were found to be MBL producers out of the 362 Gram-negative isolates recovered: similar to the results of previous study [15], which also reported the MBL production among non-fermentative bacteria only with no case of other members from *Enterobacteriaceae* showing MBL production. However, two different studies conducted in India reported production of MBL by members of *Enterobacteriaceae* including *E. coli*, *Klebsiella* spp., *Enterobacter* spp., and *Citrobacter* spp. [21, 22].

The highest prevalence rate of MBL producers in this study, was detected from urine and pus sample i.e. 7/21 (33.3%) of the total MBL producers isolated. All the MBL producing *P. aeruginosa* were isolated from urine and maximum MBL producing *Acinetobacter* spp. 7/21 (33.3%) were isolated from pus. Meanwhile, blood samples were not considered for present surveillance study owing to the lack of BACTEC™ instrumented culture system in the hospital and also because blood culture is recommended mostly for cases related to enteric fever complaints or those presenting with symptoms of sepsis which upon culture yield *Salmonella* spp. or Gram positive cocci in most of the attempts.

A PCR based method is usually considered to be the best method for detecting MBL-producing isolates. However, the increasing number of types of MBLs is creating difficulties in detection of MBLs, since primers used for PCR are usually designed to detect a single gene type [14]. Furthermore, for a low income county like Nepal use of PCR based technique in surveillance process would be expensive and undesirable from financial aspect to test every single suspected isolate. To circumvent this problem we have used phenotypic detection technique using imipenem-EDTA combined disk method, which in one hand has sensitivity and specificity of 100 and 98%, respectively and on the other hand is cost effective as well [16].

With respect to antimicrobial susceptibilities, 159 isolates i.e., 100 isolates from the member of *Enterobacteriaceae* family, 24 *P. aeruginosa* and 35 *Acinetobacter* spp. were found to be resistant against ceftazidime upon initial screening. Among the ceftazidime resistant isolates belonging to *Enterobacteriaceae* family, the most sensitive antibiotic was Nitrofurantoin, 91% followed by amikacin i.e. 85%. Polymxin B was found to be the most effective drug with 100% susceptibility to cefatzidime resistant *P. aeruginosa* followed by imipenem i.e., 91.67% which is in concordance with the findings of Mishra et al. [15]. In this study, 14/24 (58.33%) of *P. aeruginosa* were found to be resistant to Piperacillin which is in agreement with 69.1% resistance of *P. aeruginosa* to Piperacilin as reported by Aibinu et al. [23]. CLSI recommends ofloxacin as supplemental when the *P. aeruginosa* is isolated from urine sample but for the uniformity, in this study it was used to every isolates of *P. aeruginosa* irrespective of the sample type.

Likewise, among the ceftazidime resistant *Acinetobacter* spp., tigecycline was found to be the most sensitive drug i.e. 30/35 (85.7%), which is in agreement to the findings of Mishra et al. [15]. Since Tigecycline disk diffusion breakpoint for *Acinetobacter* spp. is not recommended by CLSI, for the purpose of this study, U.S. Food and drug administration (FDA) tigecycline break point criteria for *Enterobacteriaceae* i.e. ≥19 mm for susceptibility and ≤14 mm for resistance was used which in recent years have been followed by other researchers as well [24]. In this study, 24/35 (68.5%) of the ceftazidime resistant *Acinetobacter* spp. were sensitive to Imipenem. Similar reports of higher sensitivity of Imipenem to *Acinetobacter* spp. were reported in other studies [25, 26].

The highest rate of MDR in our study was seen among *E. coli* strains followed by *Acinetobacter* spp. and *P. aeruginosa*. Among the total *P. aeruginosa* isolates 44.4% were found to be MDR which is similar to 49.8% MDR reported by Strateva et al. [27]. However, some other studies conducted in Nepal have reported slightly higher MDR cases i.e. 51.3% and 65.3%, respectively [15, 28]. Likewise, among the total *Acinetobacter* spp. isolated, 82.7% were found to be MDR which is similar to previous study conducted in Nepal, where 95% of *Acinetobacter* spp. isolated were reported to be MDR [15]. Similarly, in another study conducted at National Institute of Neurological and Allied sciences, Kathmandu, 85.4% isolates of *Acinetobacter* spp. were reported MDR [28]. The current findings is an alarming sign, since almost half of the MBL producers have been found to be MDR strains leaving the medical practisoners with limited therapeutic options to combat such pathogens.

The armamentarium against MDR Gram-negative microorganisms has almost been exhausted especially after the advent of carbapenem resistance among them. Until last year, parenteral colistin available as colistin methanesulfonate (CMS) showing potent activity in vitro against MDR nosocomial *P. aeruginosa, Acinetobacter* spp., *Stenotrophomonas maltophilia*, *Enterobacter* spp. and *Klebsiell*a spp., including ESBL and carbapenemase-producers [29, 30] was the ultimate treatment option. However, recent reporting of plasmid-mediated colistin resistance in *Escherichia coli* isolated from animals, food, and patients in China by Liu et al. in November, 2016 [31], has left us with no option. Hence, proper implementation of infection control strategy, active antimicrobial stewardship approach, improved laboratory detection, judicious use of antimicrobial agents, along with regular national level surveillance can be some of the arbitration measures to control as well as aiding formulation of strategy in tackling drug resistance issues like the current one under discussion.

## Conclusion

The findings of our study demonstrated a higher prevalence of MDR and MBL positive *P. aeruginosa* and *Acinetobacter* spp., which have been globally incriminated with adverse clinical outcome including a higher morbidity and mortality rate. The study results demonstrate the serious therapeutic and epidemiological threat of the spread of metallo-beta-lactamase producers. Since, antimicrobial resistance is a growing threat worldwide with increasing resistance to third generation cephalosporins becoming a cause of concern among *Enterobacteriaceae*; early detection and infection control practices are the best defense against these organisms.

## Abbreviations

EDTA: ethylenediamine-tetraacetic acid
MBLs: metallo-beta-lactamases
GNRs: gram negative rods
CLSI: Clinical and Laboratory Standards Institute
CD: combined disk
MDR: multi drug resistance
CSF: cerebro-spinal fluid
ATCC: American type culture collection
ZOI: zone of inhibition
CAZ: cetazidime
IPM: imipenem
MEM: meropenem
NHR: Cnepal Health Research Council
OPD: outdoor patients
KPC: *Klebsiella pneumoniae* carbapenemases
LRT: nlower respiratory tract
PCR: polymerase chain reaction
FDA: food and drug administration
